# Actionable mutations in plasma cell-free DNA in patients with advanced cancers referred for experimental targeted therapies

**DOI:** 10.18632/oncotarget.3373

**Published:** 2015-02-25

**Authors:** Filip Janku, Philipp Angenendt, Apostolia M. Tsimberidou, Siqing Fu, Aung Naing, Gerald S. Falchook, David S. Hong, Veronica R. Holley, Goran Cabrilo, Jennifer J. Wheler, Sarina A. Piha-Paul, Ralph G. Zinner, Agop Y. Bedikian, Michael J. Overman, Bryan K. Kee, Kevin B. Kim, E. Scott Kopetz, Rajyalakshmi Luthra, Frank Diehl, Funda Meric-Bernstam, Razelle Kurzrock

**Affiliations:** ^1^ Department of Investigational Cancer Therapeutics (Phase I Clinical Trials Program), The University of Texas MD Anderson Cancer Center, Houston, TX, USA; ^2^ Sysmex Inostics GmbH, Hamburg, Germany; ^3^ Department of Melanoma Medical Oncology, The University of Texas MD Anderson Cancer Center, Houston, TX, USA; ^4^ Department of Gastrointestinal Medical Oncology, The University of Texas MD Anderson Cancer Center, Houston, TX, USA; ^5^ Molecular Diagnostic Laboratory, The University of Texas MD Anderson Cancer Center, Houston, TX, USA; ^6^ Moores Cancer Center, The University of California San Diego, La Jolla, CA, USA

**Keywords:** EGFR, BRAF, KRAS, PIK3CA, cell-free DNA

## Abstract

Cell-free (cf) DNA in the plasma of cancer patients offers an easily obtainable source of biologic material for mutation analysis. Plasma samples from 157 patients with advanced cancers who progressed on systemic therapy were tested for 21 mutations in *BRAF, EGFR, KRAS*, and *PIK3CA* using the BEAMing method and results were compared to mutation analysis of archival tumor tissue from a CLIA-certified laboratory obtained as standard of care from diagnostic or therapeutic procedures. Results were concordant for archival tissue and plasma cfDNA in 91% cases for *BRAF* mutations (kappa = 0.75, 95% confidence interval [CI] 0.63 – 0.88), in 99% cases for *EGFR* mutations (kappa = 0.90, 95% CI 0.71– 1.00), in 83% cases for *KRAS* mutations (kappa = 0.67, 95% CI 0.54 – 0.80) and in 91% cases for *PIK3CA* mutations (kappa = 0.65, 95% CI 0.46 – 0.85). Patients (*n* = 41) with > 1% of *KRAS* mutant cfDNA had a shorter median survival compared to 20 patients with </= 1% of *KRAS* mutant DNA (4.8 vs. 7.3 months, *p* = 0.008). Similarly, 67 patients with > 1% of mutant cfDNA (*BRAF, EGFR, KRAS*, or *PIK3CA*) had a shorter median survival compared to 33 patients with </= 1% of mutant cfDNA (5.5 vs. 9.8 months, *p* = 0.001), which was confirmed in multivariable analysis.

## INTRODUCTION

The discovery of oncogenic mutations has expanded our understanding of the mechanisms of tumorigenesis and led to the development of targeted cancer therapies directed at specific druggable targets. [1–5] Examples include BRAF inhibitors in melanoma harboring *BRAF* mutations, ABL kinase inhibitors in chronic myelogenous leukemia with *BCR-ABL* fusion, EGFR tyrosine kinase inhibitors in non-small cell lung cancer (NSCLC) with an *EGFR* mutation, and others. [1–10] Currently, oncogenic mutations are tested using archival formalin-fixed, paraffin-embedded tumor tissue (FFPE) and its lack of availability is often a limiting factor, precluding mutation analysis. In addition, mutation analysis of primary tumor tissue or of an isolated metastasis does not, due to tumor heterogeneity, necessarily reflect the genetic make-up of metastatic disease. [11–15] It has been reported that distinct oncogenic mutations occur in different areas of a primary tumor and that there is a discrepancy in approximately 20–30% of cases between the mutation status in primary tumor versus distant metastases. [11, 12] In addition, translational studies in *EGFR*-mutated NSCLC suggested that the cancer genotype can change over time. [13] Sequist et al. demonstrated, in a group of 37 patients with *EGFR*-mutant NSCLC who had pre-treatment and post-progression tumor biopsies, that some mutations occur and disappear over time. [13] For example, patients who initially responded to an *EGFR* tyrosine kinase inhibitor developed an *EGFR* T790M mutation or *PIK3CA* mutation at the time of disease progression. Consequently, their treatment regimens were changed and the *EGFR* T790M and *PIK3CA* mutations were no longer detectable in the tumor samples collected a couple of months later, and patients responded again to retreatment with an EGFR tyrosine kinase inhibitor. [13] Having a technique available to elucidate molecular changes potentially underlying drug resistance is of special importance as most patients treated with matched targeted therapies, despite improved response rates and longer progression-free survival, ultimately develop therapeutic resistance and disease progression.

Cell-free (cf) DNA is released to the circulation from cells undergoing apoptosis or necroptosis in primary or metastatic cancer lesions or in the tumor microenvironment and can be identified in the blood samples of patients with cancer. [16] Unlike performing tissue biopsies, obtaining samples of plasma cfDNA is a noninvasive approach, with less risk to patients at a lower cost. Therefore, in patients with advanced cancer, we investigated whether mutation analysis of plasma-derived cfDNA has an acceptable level of concordance with routine clinical mutation analysis for common oncogenic mutations in *BRAF*, *EGFR*, *KRAS*, and *PIK3CA.* Tissue testing obtained from prior surgeries and biopsies was performed in the Clinical Laboratory Improvement Amendment (CLIA)–certified Molecular Diagnostic Laboratory at The University of Texas MD Anderson Cancer Center (MD Anderson).

## RESULTS

### Patients

A total of 157 patients with diverse advanced cancers with known FFPE tumor tissue mutation status for mutations in at least one of the selected cancer genes, which included *BRAF*, *EGFR*, *KRAS*, *PIK3CA*, were enrolled (Table 1). Their median age was 58 years (range, 20 to 84 years) and most patients (*n* = 118, 75%) were white and men (*n* = 81, 52%). The most common tumor types were colorectal cancer (*n* = 68, 43%), melanoma (*n* = 34, 22%), NSCLC (*n* = 13, 8%), appendiceal cancer (*n* = 5, 3%), ovarian cancer (*n* = 5, 3%) and uterine cancer (*n* = 5, 3%) (Table 2). The median time between FFPE tumor tissue and plasma collection was 16.5 months (range, 0–144. 7 months). Table 3 provides information about experimental therapies that were given.

**Table 1:** Mutations tested in cfDNA and concordance between tumor tissue and cfDNA

Mutations tested		
**Gene**	Exon	Mutation type
***BRAF***	15	V600E
		V600K
***EGFR***	19	Δ E746_A750 (2235_2249del15)
		Δ E746_A750 (2235_2250del15)
		Δ E746_S752 ins V
		Δ L747_A750 ins P
		Δ L747_T751
		Δ L747_P753 ins S
	20	T790M
	21	L858R
***KRAS***	2	G12S
		G12R
		G12C
		G12D
		G12A
		G12V
		G13D
***PIK3CA***	9	E542K
		E545K
	20	H1047R
		H1047L
Concordance between mutation testing of tumor tissue and cfDNA		
**TESTED (*N* = 137)**	***BRAF* mutation in tumor**	***BRAF* wild-type in tumor**
***BRAF* mutation in cfDNA**	29	4
***BRAF* wild-type in cfDNA**	9	95
**Observed agreements**	124 (91%); Kappa 0.75, SE 0.06; 95 CI% 0.63–0.88	
**Sensitivity**	76% (95% CI 0.60–0.89)	
**Specificity**	96% (95% CI 0.90–0.99)	
**Positive predictive value**	88% (95% CI 0.72–0.97)	
**Negative predictive value**	91% (95% CI 0.84–0.96)	
**TESTED (*N* = 79)**	***EGFR* mutation in tumor**	***EGFR* wild-type in tumor**
***EGFR* mutation in cfDNA**	5	1
***EGFR* wild-type in cfDNA**	0	73
**Observed agreements**	78 (99%); Kappa 0.90, SE 0.10; 95 CI% 0.71–1.00	
**Sensitivity**	100% (95% CI 0.48–1.00)	
**Specificity**	99% (95% CI 0.93–1.00)	
**Positive predictive value**	83% (95% CI 0.36–0.97)	
**Negative predictive value**	100% (95% CI 0.95–1.00)	
**TESTED (*N* = 121)**	***KRAS* mutation in tumor**	***KRAS* wild-type in tumor**
***KRAS* mutation in cfDNA**	49	8
***KRAS* wild-type in cfDNA**	12	52
**Observed agreements**	101 (83%); Kappa 0.67, SE 0.07; 95 CI% 0.54–0.80	
**Sensitivity**	80% (95% CI 0.68–0.89)	
**Specificity**	87% (95% CI 0.75–0.94)	
**Positive predictive value**	86% (95% CI 0.74–0.94)	
**Negative predictive value**	81% (95% CI 0.70–0.90)	
**TESTED (*N* = 107)**	***PIK3CA* mutation in tumor**	***PIK3CA* wild-type in tumor**
***PIK3CA* mutation in cfDNA**	12	8
***PIK3CA* wild-type in cfDNA**	2	85
**Observed agreements**	97 (91%); Kappa 0.65, SE 0.10; 95 CI% 0.46–0.85	
**Sensitivity**	86% (95% CI 0.57–0.98)	
**Specificity**	91% (95% CI 0.84–0.96)	
**Positive predictive value**	60% (95% CI 0.36–0.81)	
**Negative predictive value**	98% (95% CI 0.92–1.00)	

**Table 2:** Clinical characteristics of 157 patients with advanced cancers

Parameter	Value
**Age**	
Median age (range)	58 (20–84)
**Gender**	
Men (%)	81 (52)
Women (%)	76 (48)
**Ethnicity**	
White (%)	118 (75)
African-American (%)	20 (13)
Hispanic (%)	15 (10)
Asian (%)	4 (3)
**Tumor type**	
Colorectal cancer (%)	68 (43)
Melanoma (%)	34 (22)
Non-small cell lung cancer (%)	13 (8)
Ovarian cancer (%)	5 (3)
Appendiceal cancer (%)	5 (3)
Uterine cancer (%)	5 (3)
Breast cancer (%)	4 (3)
Non-squamous head and neck cancer (%)	4 (3)
Gastroesophageal cancer (%)	3 (2)
Papillary thyroid cancer (%)	3 (2)
Prostate cancer (%)	2 (2)
Soft tissue sarcoma (%)	2 (2)
Ampullary cancer (%)	1 (<1)
Cholangiocarcinoma (%)	1 (<1)
Merkel cell cancer (%)	1 (<1)
Small cell lung cancer (%)	1 (<1)
Carcinoma of unknown primary (%)	1 (<1)
Duodenal cancer (%)	1 (<1)
Hepatocellular carcinoma (%)	1 (<1)
Squamous head and neck cancer (%)	1 (<1)
Erdheim-Chester disease (%)	1 (<1)

**Table 3:** Experimental therapies in patients with *BRAF*, *EGFR*, *KRAS* and *PIK3CA* mutations

Mutation	Total	Matched therapy	Non-matched therapy	No therapy
***BRAF* (tumor)**	38	33^1^	2	3
***BRAF* (cfDNA)**	33	29^1^	1	3
***EGFR* (tumor)**	5	4^2^	1	0
***EGFR* (cfDNA)**	6	4^2^	2	0
***KRAS* (tumor)**	61	0^3^	47	14
***KRAS* (cfDNA)**	57	0^3^	43	14
***PIK3CA* (tumor)**	14	9^4^	4	1
***PIK3CA* (cfDNA)**	20	8^4^	9	3

1 BRAF and MEK inhibitors were considered as matched therapies

2 EGFR inhibitors were considered as matched therapies

3 There were no matched therapies

4 PI3K/AKT/mTOR inhibitors were considered as matched therapies

### Mutations and discrepancy analysis

Of the 157 patients, 137 were tested for *BRAF* mutation in tumor and cfDNA samples; 38 (28%) patients had a *BRAF* V600 mutation in the FFPE tumor samples and 33 (24%) had *BRAF* V600 mutations in cfDNA from plasma, with overall agreement between testing modalities in 124 (91%) cases (kappa 0.75, SE 0.07, 95% confidence interval [CI] 0.63–0.88) with sensitivity 76% (95% CI 0.60–0.89), specificity 96% (95% CI 0.90–0.99), positive predictive value 88% (95% CI 0.72–0.97) and negative predictive value 91% (95% CI 0.84–0.96; Table 1). Of 9 patients (melanoma, *n* = 6; colorectal, *n* = 1; papillary thyroid, *n* = 1; appendiceal cancer, *n* = 1) with *BRAF* V600E mutation in the tumor tissue but not in cfDNA, one patient (papillary thyroid cancer) had wt*BRAF* when the tumor tissue (we used the same block as used for initial tissue testing, if possible) was tested with BEAMing. Three patients (all melanoma) had plasma collection immediately after coming off BRAF or MEK targeted therapy and 1 patient (appendiceal cancer) had plasma collection right after being taken off standard chemotherapy without evidence of disease progression. Of interest, the latter patient had another cfDNA analysis for a *BRAF* mutation when her disease progressed and at that time *BRAF* V600E-mutant cfDNA was detected. In addition, a patient with colorectal cancer and a *BRAF* V600E mutation in the tumor tissue, but not in cfDNA, was found to have a *KRAS* G12D mutation in cfDNA, which was not detected in the tumor tissue. Furthermore, we found a *BRAF* V600K mutation in cfDNA in 4 patients with wt *BRAF* in their tumor tissue. Of interest, 2 of these 4 patients (colorectal cancer and NSCLC) also had *KRAS* G12 mutations in FFPE tumor samples and cfDNA. Finally, a patient with melanoma had a *BRAF* V600E mutation in the tissue, but a *BRAF* V600K mutation in cfDNA; however, repeated testing of tumor tissue with BEAMing confirmed a *BRAF* V600K mutation. We also analyzed whether the amount of *BRAF*-mutant cfDNA correlated with discrepancies between cfDNA and tumor tissue and, indeed, patients with concordant results between cfDNA and tissue had a median of 1.99% of *BRAF*-mutant cfDNA compared to a median of 0.02% of *BRAF*-mutant cfDNA in patients with discrepant results (*p* = 0.001).

Of the 157 patients, 79 were tested for *EGFR* in tumor and cfDNA samples; 5 (6%) patients had *EGFR* mutations in the FFPE tumor samples and 6 (8%) had *EGFR* mutations in cfDNA from plasma, with overall agreement between testing in 78 (99%) cases (kappa = 1.00, SE 0.10, 95% CI 0.71–1.00) with sensitivity 100% (95% CI 0.48–1.00), specificity 99% (95% CI 0.93–1.00), positive predictive value 83% (95% CI 0.36–0.97) and negative predictive value 100% (95% CI 0.95–1.00; Table 1). A patient with Erdheim-Chester disease had an *EGFR* T790M mutation not previously identified in FFPE tumor samples. Furthermore, 2 patients with *EGFR*-mutant NSCLC previously treated with EGFR tyrosine kinase inhibitors also had a second *EGFR* T790M mutation not previously identified in the FFPE tumor samples, which can plausibly explain why secondary resistance to EGFR targeted therapies occurred. Of interest, a patient treated with erlotinib, who had a biopsy at the time of disease progression revealing an *EGFR* exon 19 deletion and *EGFR* T790M mutation, demonstrated no *EGFR* T790M mutation in cfDNA after 10 months of being taken off of erlotinib therapy. Because of the low number of patients with *EGFR* mutations, we did not perform analysis to test associations between the amount of mutant cfDNA and the rate of discrepancies (tumor tissue vs. cfDNA).

Of the 157 patients, 121 were tested for *KRAS* in tumor and cfDNA samples; 61 (50%) patients had *KRAS* G12 or 13 mutations in the FFPE tumor samples and 57 (47%) had *KRAS* mutations in cfDNA from plasma with overall agreement between testing in 101 (83%) cases (kappa = 0.67, SE 0.07, 95% CI 0.54–0.80) with sensitivity 80% (95% CI 0.68–0.89), specificity 87% (95% CI 0.75–0.94), positive predictive value 86% (95% CI 0.74–0.94) and negative predictive value 81% (95% CI 0.70–0.90; Table 1). Of 12 patients (colorectal, *n* = 6; appendiceal cancer, *n* = 2; NSCLC, *n* = 2; duodenal, *n* = 1; breast cancer, *n* = 1) who had *KRAS* G12 or G13 mutations in the tumor tissue did not have these mutations in their cfDNA. In addition, of 8 patients (colorectal cancer, *n* = 3; NSCLC, *n* = 1; endometrial cancer, *n* = 1; breast cancer, *n* = 1; ovarian cancer, *n* = 1; melanoma, *n* = 1) with *KRAS* G12 or G13 mutation in cfDNA, but not in FFPE tumor samples, 2 patients had *KRAS* Q61 mutations in FFPE. We also analyzed whether the amount of *KRAS*-mutant cfDNA correlated with discrepancies between cfDNA and tissue. Patients with concordant results between cfDNA and tissue had a median of 7.46% *KRAS*-mutant cfDNA compared to a median of 0.55% *KRAS*-mutant cfDNA in patients with discrepant results (*p* = 0.048).

Of the 157 patients, 107 were tested for *PIK3CA* in tumor and cfDNA samples; in 14 (13%) patients *PIK3CA* mutations were detected in FFPE tumor samples and 20 (19%) had *PIK3CA* mutations in cfDNA from plasma with overall agreement between testing in 97 (91%) cases (kappa = 0.65, SE 0.10, 95% CI 0.46–0.85) with sensitivity 86% (95% CI 0.57–0.98), specificity 91% (95% CI 0.84–0.96), positive predictive value 60% (95% CI 0.36–0.81) and negative predictive value 98% (95% CI 0.92–1.00; Table 1). Two patients (breast cancer, *n* = 1; NSCLC, *n* = 1) had a *PIK3CA* H1047R mutation their FFPE tumor samples, but not in cfDNA. In contrast, 8 patients (colorectal cancer, *n* = 4; squamous cell head and neck, *n* = 1; non-squamous head and neck cancer, *n* = 1; breast, *n* = 1; NSCLC, *n* = 1) had *PIK3CA* E542K or E545K mutations in cfDNA but not in FFPE tumor samples. Of interest, 3 of these patients (head and neck, *n* = 2; NSCLC, *n* = 1) were also known to have *EGFR* mutations and progressed on an EGFR monoclonal antibody or tyrosine kinase inhibitor, suggesting that a *PIK3CA* mutation could have been a driver of therapeutic resistance. In addition, 2 patients (both with colorectal cancer) had a different *PIK3CA* mutation in the FFPE tumor samples (Q546P and E545D/M1043L, which were not included in the BEAMing panel). Finally, 4 patients with *PIK3CA* mutations in cfDNA, but not FFPE tumor samples, had simultaneous *KRAS* mutations in cfDNA and FFPE tumor samples. We also analyzed whether the amount of *PIK3CA*-mutant cfDNA correlated with discrepancies between cfDNA and tumor tissue and patients with concordant results in cfDNA and tumor tissue had a median of 1.83% of *PIK3CA*-mutant cfDNA compared to a median of 2.61% of *PIK3CA*-mutant cfDNA in patients with discrepant results (*p* = 0.50).

### Emergence of low frequency mutations in cfDNA

In several patients, testing of cfDNA revealed mutations not previously detected in the tumor tissue, which in some of them could have plausibly explained resistance to pertinent targeted therapies. For instance, a patient with NSCLC with wt *BRAF* and a *KRAS* G12C mutation in the tissue and cfDNA (3.80%) was also found to have a low frequency *BRAF* V600K mutation in cfDNA (0.03) at the time of disease progression while taking a MEK inhibitor for 3.7 months. A patient with colorectal cancer with wt *BRAF* and wt *KRAS* in the primary tumor, who received a cetuximab-based combination for nearly one year had an emergence of a low frequency *BRAF* V600K mutation in cfDNA (0.02%).

Furthermore, a patient with NSCLC and an *EGFR* L858R mutation found in an original tumor biopsy had cfDNA collection after developing secondary resistance to the EGFR tyrosine kinase inhibitor erlotinib, which in addition to a known *EGFR* L858R mutation (0.11%), revealed an *EGFR* T790M mutation (0.04%), plausibly explaining secondary resistance to erlotinib. A patient with NSCLC and an *EGFR* exon 19 deletion from the original biopsy had cfDNA collected after becoming refractory to erlotinib; cfDNA revealed, in addition to an *EGFR* exon 19 deletion (6.42%), *EGFR* T790M (0.65%) and *PIK3CA* E545K (0.67%) mutations not previously identified in the earlier tissue testing, which can credibly explain the emergence of resistance. A patient with NSCLC and an *EGFR* exon 19 deletion was also found to have a simultaneous *EGFR* T790M mutation on a tumor biopsy obtained after progression while taking the EGFR tyrosine kinase inhibitor erlotinib; however, unlike with the *EGFR* exon 19 deletion (12.86%), *EGFR* T790M was no longer present in cfDNA obtained 10 months after having been taken off erlotinib.

Furthermore, a patient with a *BRAF* V600E-mutant, wt *KRAS* colorectal cancer, with a history of early progression to cetuximab-based therapy, was found to have a *KRAS* G12D mutation (24.39%) in cfDNA instead, which was not previously detected in the tumor tissue. A patient with wt *KRAS* in the initial tumor tissue biopsy who had a transient response (4 months) to cetuximab-based therapy was then found to have a *KRAS* G13D mutation (0.88%) in cfDNA. Finally, a patient with ovarian cancer and a *PIK3CA* H1047R mutation in an original FFPE tumor sample, who had dramatic but short-lived response to an investigational agent targeting PI3K alpha, was found, in addition to having a *PIK3CA* H1047R mutation (0.08%), a low frequency *KRAS* G12C mutation (0.03%) in cfDNA from plasma collected before initiation of a PI3K inhibitor, which can reasonably explain early therapeutic failure. [21, 22]

Further, a patient with mucoepidermoid carcinoma of the nasal-lacrimal sac with wt *PIK3CA* and an *EGFR* exon 18 mutation (A722V) on an initial biopsy was also found to have a *PIK3CA* E545K mutation in cfDNA, and the patient was ultimately refractory to treatment with the EGFR tyrosine kinase inhibitor erlotinib. Also, a patient with squamous cell carcinoma of head and neck with wt *PIK3CA* and an *EGFR* exon 21 mutation (H835L) in an initial resected tumor was found to have a *PIK3CA* E545K mutation (0.05%) in cfDNA collected after progressive disease following 3 months of cetuximab, carboplatin and paclitaxel treatment.

### Mutations in cfDNA and overall survival

Next we investigated whether the amount of mutant cfDNA (percentage compared to wt) had any impact on overall survival (OS). Our strategy was to compare patients with </= 1% of mutant cfDNA vs. > 1% cfDNA to make comparable categories. These thresholds were selected based on a 5% trimmed mean value of mutated cfDNA for all tested genes, which was deemed to be more representative since it was not affected by the number of patients without cfDNA mutations. In addition, these thresholds reflect approximate medians of the percent of mutant DNA for *BRAF*, *EGFR*, *KRAS* and *PIK3CA* (1%, 2.7%, 3.8% and 0.5%, respectively).

**Figure 1:**
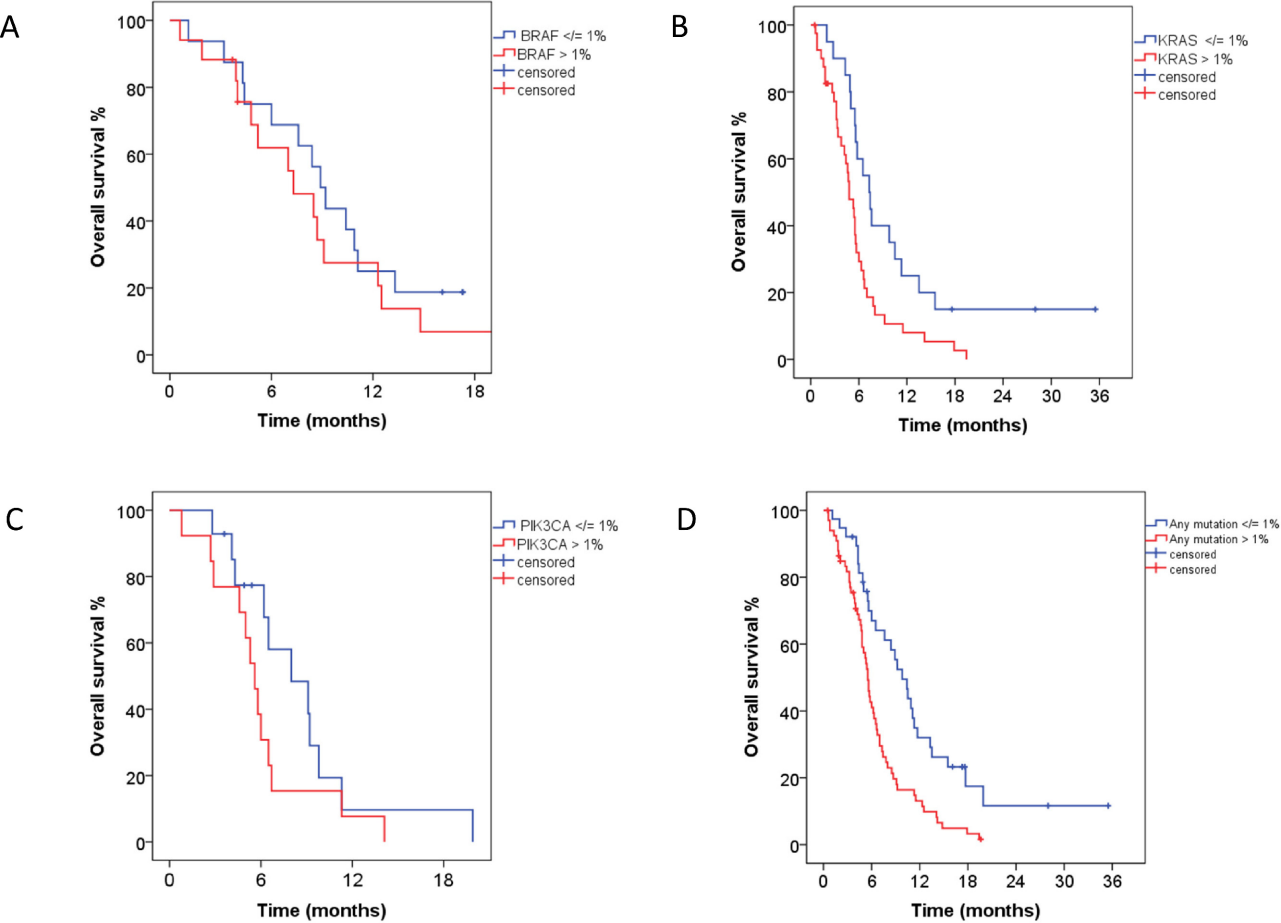
**(A)** In 33 patients with *BRAF* mutations in cfDNA, 16 patients with </= 1% (blue) of *BRAF* mutations had survival similar to 17 patients with > 1% (red) of *BRAF* mutations (8.9 months, 95% CI 7.3–10.5 vs. 7.3 months, 95% CI 4.5–10.1; *p* = 0.38). **(B)** In 61 patients with *KRAS* mutations in cfDNA, 20 patients with </= 1% (blue) of *KRAS* mutations had longer median survival compared to 41 patients with > 1% (red) of *KRAS* mutations (7.3 months, 95% CI 5.3–9.3 vs. 4.8 months, 95% CI 3.8–5.8; *p* = 0.008). **(C)** In 27 patients with *PIK3CA* mutations in cfDNA, 14 patients with </= 1% (blue) of *PIK3CA* mutations had survival similar to 13 patients with > 1% (red) of *PIK3CA* mutations (8.0 months, 95% CI 4.0–12.0 vs. 5.6 months, 95% CI 4.7–6.5; *p* = 0.15). **(D)** In 105 patients with *BRAF*, *EGFR*, *KRAS*, or *PIK3CA* mutations in cfDNA, 38 patients with </= 1% (blue) of mutant cfDNA had longer median survival compared to 67 patients with > 1% (red) of mutant cfDNA (9.8 months, 95% CI 7.5–12.1 vs. 5.5 months, 95% CI 5.0–6.0; *p* = 0.001).

In 33 patients with *BRAF* mutations in cfDNA, 16 patients with </= 1% of *BRAF*-mutant cfDNA had survival rates similar to 17 patients with > 1% of *BRAF*-mutant cfDNA (8.9 months, 95% CI 7.3–10.5 vs. 7.3 months, 95% CI 4.5–10.1; *p* = 0.38; Figure 1A). Of interest, 20 patients with </= 1% of *KRAS*-mutant cfDNA had a longer median survival compared to 41 patients with > 1% of *KRAS*-mutant cfDNA (7.3 months, 95% CI 5.3–9.3 vs. 4.8 months, 95% CI 3.8–5.8; *p* = 0.008; Figure 1B). Finally, 14 patients with </= 1% of *PIK3CA*-mutant cfDNA had a similar length of survival as did 13 patients with > 1% of *PIK3CA*-mutant cfDNA (8.0 months, 95% CI 4.0–12.0 vs. 5.6 months, 95% CI 4.7–6.5; *p* = 0.15; Figure 1C). Survival analysis for patients with *EGFR* mutations has not been performed due to the low number of patients in that group.

Next, we performed an analysis combining all tested mutations (*BRAF*, *EGFR*, *KRAS*, *PIK3CA*) in all of 105 patients with mutant cfDNA. When there was more than one mutation in the same patient, the mutation with the highest percentage of mutant DNA was used for analysis. Patients (*n* = 38) with </= 1% of mutant cfDNA had longer median survivals compared to 67 patients with > 1% of mutant cfDNA (9.8 months, 95% CI 7.5–12.1 vs. 5.5 months, 95% CI 5.0–6.0; *p* = 0.001; Figure 1D).

Finally, we analyzed the prognostic impact of cfDNA mutations on OS in multivariable analysis, which included the Royal Marsden Hospital prognostic score (RMH score) and the MD Anderson (MDACC) score. [23, 24] The RMH score is a prospectively validated tool used to predict OS in patients with advanced cancers referred for early-phase clinical trials. It is calculated on the basis of lactate dehydrogenase levels (> upper limit of normal vs. normal), albumin levels (<3.5 g/mL vs. 3.5 g/mL or higher) and number of metastatic sites (> 2 sites vs. 2 sites or less) and scores of 0–1 are associated with better survival than scores of 2–3. Similarly, the MDACC prognostic score included the factors listed above for the RMH score as well as ECOG performance status (0 vs. >/=1) and type of tumor (gastrointestinal vs. other).

**Figure 2:**
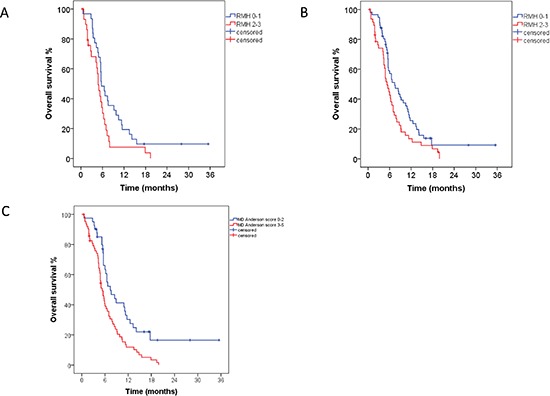
**(A)** In 61 patients with *KRAS* mutations in cfDNA, 31 patients with scores of 0–1 had longer median survival than 30 patients with RMH scores of 2–3 (5.7 months, 95% CI 4.4–7.0 vs. 4.8 months, 95% CI 3.9–5.7; *p* = 0.036). **(B)** In a combined analysis of 105 patients with any cfDNA mutation, 57 patients with RMH scores of 0–1 had longer median survival than did 48 patients with RMH scores of 2–3 (7.4 months, 95% CI 4.9–9.9 vs. 5.3 months, 95% CI 4.2–6.4; *p* = 0.029). **(C)** In a combined analysis of 105 patients with any cfDNA mutation, 41 patients with MDACC scores of 0–2 had longer median survival than 64 patients with MDACC scores of 3–5 (7.4 months, 95% CI 4.5–10.3 vs. 5.3 months, 95% CI 4.3–6.3; *p* = 0.002).

In 61 patients with *KRAS* mutations in cfDNA, 31 patients with RMH scores of 0–1 had longer median survivals than 30 patients with RMH scores of 2–3 (5.7 months, 95% CI 4.4–7.0 vs. 4.8 months, 95% CI 3.9–5.7; *p* = 0.036, Figure 2A). In multivariable analysis, which included *KRAS* mutations in cfDNA (</= 1% vs. > 1%) and the RMH score (0–1 vs. 2–3), patients with *KRAS* mutations in </= 1% of cfDNA had a trend toward a longer survival compared to patients with *KRAS* mutations in > 1% of cfDNA (hazard ratio [HR] 0.53, 95% CI 0.27–1.03, *p* = 0.06).

In a combined analysis of 105 patients with cfDNA mutations, 57 patients with RMH scores of 0–1 had longer median survivals than 48 patients with RMH scores of 2–3 (7.4 months, 95% CI 4.9–9.9 vs. 5.3 months, 95% CI 4.2–6.4; *p* = 0.029, Figure 2B). Similarly, 41 patients with MDACC scores of 0–2 had longer median survivals than 64 patients with MDACC scores of 3–5 (7.4 months, 95% CI 4.5–10.3 vs. 5.3 months, 95% CI 4.3–6.3; *p* = 0.002; Figure 2C). In multivariable analysis, which included mutant cfDNA (</= 1% vs. > 1%) and RMH score (0–1 vs. 2–3), patients with </= 1% of mutant cfDNA had a longer survival compared to patients with > 1% of mutant cfDNA (HR 0.49, 95% CI 0.29–0.81, *p* = 0.005). Similar results were obtained using the MDACC score (HR 0.51, 95% CI 0.32–0.82, *p* = 0.005, Table 4).

**Table 4:** Multivariable analysis of 105 patients with cfDNA mutations, which included mutant cfDNA (</= 1% vs. > 1%) and RMH score (0–1 vs. 2–3) or MDACC score (0–2 vs. 3–5)

Outcome	Variable	Hazard ratio	95% Confidence interval	*P* value
Overall survival (RMH model)	cfDNA (</= 1% vs. > 1%)	0.49	0.29–0.81	0.005
	RMH score (0–1 vs. 2–3)	0.87	0.55–1.39	0.57
Overall survival (MDACC model)	cfDNA (</= 1% vs. > 1%)	0.51	0.32–0.82	0.005
	MDACC score (0–2 vs. 3–5)	0.61	0.38–0.96	0.033

## DISCUSSION

In our study, we demonstrated that using the BEAMing technology, testing for 21 oncogenic mutations in *BRAF*, *EGFR*, *KRAS* and *PIK3CA* genes in the plasma cfDNA of patients with advanced cancers referred for treatment with experimental targeted therapies, is feasible. In addition, testing of cfDNA demonstrated acceptable concordance (*BRAF*, 91%; *EGFR*, 99%; *KRAS*, 83%; *PIK3CA*, 91%) with standard of care mutation analysis of primary or metastatic tumor tissue obtained during clinical care. Higgins et al. [17] demonstrated a 100% concordance between using BEAMing to assess *PIK3CA* mutations in plasma cfDNA versus using BEAMing for *PIK3CA* mutations in the tumor tissue in a cohort of patients with advanced breast cancer when the plasma and tumor samples were obtained at the same time. However, the concordance decreased to 79% when tumor samples and plasma cfDNA were obtained at different time points. Board et al. [24] demonstrated a 95% concordance between *PIK3CA* mutation status in plasma cfDNA and tumor tissue obtained at the same time by using an amplification refractory mutation system. Most recently, Thierry et al. [25] demonstrated a 96% concordance for combined *KRAS* and *BRAF* mutation testing using allele-specific quantitative PCR of plasma cfDNA compared to tissue (primary or metastatic) tested as standard of care. It is conceivable that mutation analysis results from cfDNA are highly concordant with mutation analysis results from tumor tissue if both materials are obtained concomitantly. However, the concordance rate can decrease, perhaps due to inherent heterogeneity, if both materials are obtained at different time points. This is not unexpected, since similar observations were made in a study in 33 matched primary and recurrent breast tumors, in which 97 of 112 (86.6%) somatic mutations were concordant. [26] It is unclear, why our results demonstrated the lowest concordance for *KRAS* compared to other genes (83% vs 91%–99%) and whether this was related to underlying biology or technology (or both).

Detection of molecular aberrations in cfDNA can be also used to monitor response to therapy and emergence of secondary mutations associated with resistance to therapy, which can plausibly be used for therapeutic interventions. [27, 28] Because plasma cfDNA can originate from multiple tumor sites, arguably its molecular analysis may better reflect prevailing molecular aberrations than obtained from single-site biopsied tissue. [29, 30] In addition, unlike tissue biopsies, obtaining samples of cfDNA is a noninvasive approach, with less risk to patients at a lower cost. Diehl et al. [16], in a pilot study of 18 patients with metastatic colorectal cancer who were indicated as being candidates for surgical resection or radiofrequency ablation, showed that cfDNA from plasma samples can be isolated and oncogenic mutations (*APC*, *KRAS*, *TP53*) can be detected in all tested patients using the BEAMing PCR-based technology. Further, analysis of a quantity of mutant copies more accurately predicted disease progression than standard evaluation of serum CEA levels. In a study of patients with metastatic breast cancer, 97% had genetic alterations in cfDNA and changes in cfDNA mutation levels correlated with changes in tumor burden to a greater degree than indicated by a CA 15–3 prognostic marker. Furthermore, two pilot studies in advanced colorectal cancer patients with wt*KRAS* demonstrated emerging *KRAS* aberrations in cfDNA during treatment with an anti-EGFR therapy [31, 32]. The first study published reported that 38% of patients treated with the anti-EGFR monoclonal antibody cetuximab, who were known to have wt *KRAS* on the basis of tumor tissue analysis, later developed *KRAS* mutations. These mutations were detectable in blood samples, usually between 5 and 6 months following treatment. [31] The second study, in patients who developed resistance to cetuximab or panitumumab, showed the emergence of *KRAS* amplification in one sample and acquisition of secondary *KRAS* mutations in 60% of the cases. *KRAS*-mutant alleles were also detectable in the blood samples of cetuximab-treated patients as early as 10 months before disease progression appeared on restaging scans. [32] In our study we did not perform serial plasma collections at multiple time points; however, we noticed several interesting observations. For instance, we found in patients with NSCLC and *EGFR* mutations in the tumor tissue and prior therapy with EGFR inhibitors, secondary mutations (*EGFR* T790M and *PIK3CA* E545K) in plasma cfDNA or *KRAS* or *BRAF* mutations in the cfDNA of patients with colorectal cancer with wt*KRAS* in tumor tissue treated with EGFR antibodies, credibly explaining adaptive resistance to therapy.

Finally, it has been suggested that the presence and amount of mutant cfDNA can be associated with progression-free and OS. [16, 28, 33, 34] For instance, in a pivotal study, the absence of cfDNA in patients with colorectal cancer after surgical resection was associated with 100% recurrence-free survival. [16] Similarly, a higher amount of cfDNA and *KRAS*-mutant cfDNA found in patients with advanced colorectal cancer treated with irinotecan and cetuximab and in patients with advanced NSCLC treated with carboplatin and vinorelbine was associated with a shorter progression-free survival and OS. [33, 34] Finally, in a group of 206 patients with metastatic colorectal cancer, a higher concentration of cfDNA negatively correlated with OS. [28] In our study, a higher percentage of mutant cfDNA, irrespective of type of the mutation, was associated with a shorter OS (7.4 months vs. 5.3 months; *p* = 0.029), which was confirmed in a multivariable analysis (HR 0.49, 95% CI 0.29–0.81, *p* = 0.005). We made a similar observation in a separate analysis of patients with *KRAS* mutations in cfDNA, which comprised the largest subgroup of our total patient population. Nevertheless, these results need to be interpreted cautiously and validated in future studies since they might have been influenced by tumor heterogeneity, the heterogeneity of our studied population and other factors.

In summary, we demonstrated that molecular analysis of cfDNA for selected oncogenic mutations in *BRAF*, *EGFR*, *KRAS*, and *PIK3CA* is feasible and demonstrates acceptable concordance with standard of care mutation testing of archival tumor tissue and that the amount of mutant cfDNA is an independent prognostic factor for survival. The possible impact of cfDNA mutations on survival must be interpreted with caution because of the retrospective nature of the study and the absence of a validation cohort. Furthermore, other factors such as tumor burden and proliferative activity were not assessed. Finally, even if a higher mutation burden predicts poor survival it remains unclear whether adding more effective therapies targeting underlying molecular aberrations and the tumor microenvironment might offset this effect. We also showed that mutations not originally found in the tumor tissue could be present at a low frequency in cfDNA, which can plausibly contribute to therapeutic resistance. In order to prove clinical utility, mutation analysis of cfDNA will need to be tested in prospective clinical trials, which will also include therapeutic intervention with respect to cfDNA mutation status. In addition, most of the sensitive technologies applicable for cfDNA testing, including BEAMing in our study, use PCR-based technologies, which limits simultaneous detection for multiple mutations. New technologies with high sensitivity and broad multiplex capability need to be developed to advance the results of analysis to the clinical arena.

## METHODS

### Patients

Starting in October 2010, patients with advanced cancers previously treated with standard therapies, who were previously tested for *BRAF* and/or *EGFR* and/or *KRAS* and/or *PIK3CA* mutations in archival tumor tissue were enrolled in the study. Patients were required to be new referrals to the Department of Investigational Cancer Therapeutics as candidates for experimental therapies or potential patients had progressive disease if already treated with experimental therapies. The registration of patients in the database, tumor pathology assessment, and tumor mutation analysis were performed at MD Anderson. The study was conducted in accordance with MD Anderson Institutional Review Board guidelines.

### Tumor tissue analyses

A total of 21 activating mutations in *BRAF*, *EGFR*, *KRAS* and *PIK3CA* genes were investigated in archival tumor tissue obtained from routine clinical diagnostic and/or therapeutic procedures from primary or metastatic sites (Table 1). All histologies were centrally reviewed at MD Anderson. Mutation testing was performed in the CLIA–certified Molecular Diagnostic Laboratory within the Division of Pathology and Laboratory Medicine at MD Anderson. DNA was extracted from microdissected paraffin-embedded tumor sections and analyzed using a polymerase chain reaction-based DNA sequencing method for mutations outlined in Table 1 utilizing primers designed by the MD Anderson Molecular Diagnostic Laboratory. In January 2011, the assay was changed to mass spectrometric detection (Sequenom MassARRAY) and in March 2012, to next-generation sequencing (Ion Torrent, Life Technologies, Carlsbad, CA). The mutations identified during the initial screening were confirmed using a Sanger sequencing. The lower limit of detection is approximately 5–10% of the mutant allele frequency, which is influenced by clonal heterogeneity and the presence of normal tissue.

### Plasma cfDNA analyses

Plasma samples used for cfDNA mutation analysis were obtained from whole blood collected in EDTA tubes, which was centrifuged and spun twice within 2 hours of collection. Isolation of cfDNA from plasma was carried out using the QIAamp DNA purification kit (Qiagen) and mutation analysis for a total of 21 mutations in *BRAF*, *EGFR*, *KRAS*, and *PIK3CA* (Table 1) with BEAMing assays were conducted on each sample by Inostics GmbH as previously published. [16–18] Briefly, individual DNA molecules were attached to magnetic beads in water- in-oil emulsions and then subjected to compartmentalized PCR amplification. The mutational status of DNA bound to beads was determined by hybridization to fluorescent allele-specific probes for mutant or wild-type (wt) of the gene of interest, respectively. Quantification of mutant DNA was performed using flow cytometry. Investigators performing mutation analysis of cfDNA with BEAMing were blinded to the results of mutation analysis of the archival tumor samples. The lower limit of detection is approximately 0.02% of mutant allele frequency.

### Statistical analysis

Concordance between mutation analysis of archival tumor tissue and mutation analysis of cfDNA from plasma samples was calculated using a kappa coefficient. Concordance analyses were carried out using GraphPad Software (GraphPad Software, Inc.; La Jolla; CA). OS was defined as the time interval from the study entry to the date of death or the date of last follow up, whichever occurred first. OS was estimated using the method of Kaplan and Meier and compared among the subgroups of patients using a log-rank test. [19] Cox proportional hazards regression models were fit to assess the association between patient characteristics and OS. [20] All tests were two-sided, and *P*-values less than 0.05 were considered statistically significant. All statistical analyses were carried out using GraphPad Software (GraphPad Software, Inc.; La Jolla; CA) and SPSS 21 computer software (SPSS Chicago, IL).
