# Erratum: Actionable mutations in plasma cell-free DNA in patients with advanced cancers referred for experimental targeted therapies

**DOI:** 10.18632/oncotarget.5663

**Published:** 2015-09-14

**Authors:** Filip Janku, Philipp Angenendt, Apostolia M. Tsimberidou, Siqing Fu, Aung Naing, Gerald S. Falchook, David S. Hong, Veronica R. Holley, Goran Cabrilo, Jennifer J. Wheler, Sarina A. Piha-Paul, Ralph G. Zinner, Agop Y. Bedikian, Michael J. Overman, Bryan K. Kee, Kevin B. Kim, E. Scott Kopetz, Rajyalakshmi Luthra, Frank Diehl, Funda Meric-Bernstam, Razelle Kurzrock

Oncotarget. 2015; 6:12809-12821

PMID: 25980577

The Abstract is incorrect in Pubmed.

Cell-free (cf) DNA in the plasma of cancer patients offers an easily obtainable source of biologic material for mutation analysis. Plasma samples from 157 patients with advanced cancers who progressed on systemic therapy were tested for 21 mutations in BRAF, EGFR, KRAS, and PIK3CA using the BEAMing method and results were compared to mutation analysis of archival tumor tissue from a CLIA-certified laboratory obtained as standard of care from diagnostic or therapeutic procedures. Results were concordant for archival tissue and plasma cfDNA in 91% cases for BRAF mutations (kappa = 0.75, 95% confidence interval [CI] 0.63 - 0.88), in 99% cases for EGFR mutations (kappa = 0.90, 95% CI 0.71- 1.00), in 83% cases for KRAS mutations (kappa = 0.67, 95% CI 0.54 - 0.80) and in 91% cases for PIK3CA mutations (kappa = 0.65, 95% CI 0.46 - 0.85). Patients (n = 41) with > 1% of KRAS mutant cfDNA had a shorter median survival compared to 20 patients with 1% of mutant cfDNA (BRAF, EGFR, KRAS, or PIK3CA) had a shorter median survival compared to 33 patients with </= 1% of mutant cfDNA (5.5 vs. 9.8 months, p = 0.001), which was confirmed in multivariable analysis.

The corrected Abstract is provided here.

Cell-free (cf) DNA in the plasma of cancer patients offers an easily obtainable source of biologic material for mutation analysis. Plasma samples from 157 patients with advanced cancers who progressed on systemic therapy were tested for 21 mutations in BRAF, EGFR, KRAS, and PIK3CA using the BEAMing method and results were compared to mutation analysis of archival tumor tissue from a CLIA-certified laboratory obtained as standard of care from diagnostic or therapeutic procedures. Results were concordant for archival tissue and plasma cfDNA in 91% cases for BRAF mutations (kappa = 0.75, 95% confidence interval [CI] 0.63 – 0.88), in 99% cases for EGFR mutations (kappa = 0.90, 95% CI 0.71– 1.00), in 83% cases for KRAS mutations (kappa = 0.67, 95% CI 0.54 – 0.80) and in 91% cases for PIK3CA mutations (kappa = 0.65, 95% CI 0.46 – 0.85). Patients (n = 41) with > 1% of KRAS mutant cfDNA had a shorter median survival compared to 20 patients with </= 1% of KRAS mutant DNA (4.8 vs. 7.3 months, p = 0.008). Similarly, 67 patients with > 1% of mutant cfDNA (BRAF, EGFR, KRAS, or PIK3CA) had a shorter median survival compared to 33 patients with </= 1% of mutant cfDNA (5.5 vs. 9.8 months, p = 0.001), which was confirmed in multivariable analysis.

